# Consensus Values, Regressions, and Weighting Factors

**DOI:** 10.6028/jres.094.020

**Published:** 1989

**Authors:** Robert C. Paule, John Mandel

**Affiliations:** National Institute of Standards and Technology, Gaithersburg, MD 20899

**Keywords:** components of variance (within- and between-groups), consensus values, convergence proof, Taylor series, weighted average, weighted least squares regression

## Abstract

An extension to the theory of consensus values is presented. Consensus values are calculated from averages obtained from different sources of measurement. Each source may have its own variability. For each average a weighting factor is calculated, consisting of contributions from both the within- and the between-source variability. An iteration procedure is used and calculational details are presented. An outline of a proof for the convergence of the procedure is given. Consensus values are described for both the case of the weighted average and the weighted regression.

## 1. Introduction

The problem of computing consensus values when the errors of measurement involve both internal (within group) and external (between group) components has been discussed in a number of papers [1–4]. The present authors have studied the case of a simple weighted average, as well as that in which the measured quantity *y* is a linear function of a known variable *x*.

In the present paper we extend our results to cases in which the error standard deviations are functions, of known form, of the *x*-variables. We also provide an outline of a proof for convergence of the iterative process described in reference [1].

While our procedure is entirely reasonable, and results in acceptable values, we have no mathematical proof that the weights, which we calculate from the data, are optimal in any well-defined theoretical sense. The problem has been recognized in the literature [5], but we know of no attempt to provide the proof of optimality.

## 2. Review

If *ω_i_* denotes the weight (reciprocal variance) of a quantity, ${\bar{Y}}_{i},$ then the general equation for a weighted average is:

$$\tilde{Y}=\frac{\sum_{i=1}^{m}\omega_{i}\;{\bar{Y}}_{i}}{\sum_{i=1}^{m}\omega_{i}}.$$ (1)

If ${\bar{Y}}_{i},$ equals the average of *n_i_* results from group *i* (*i*=1 to *m*), then

$$\mathrm{Var}\left({\bar{Y}}_{i}\right)=\frac{\sigma_{w_{i}}^{2}}{n_{i}}+\sigma_{b}^{2},$$

where

$\sigma_{w_{i}}$ = the component of standard deviation within group *i* (the $\sigma_{w_{i}}$ value can be estimated from the *n_i_* results within each group)
*σ*_b_ = the component of standard deviation between groups.

Then the weight *ω_i_* of ${\bar{Y}}_{i}$ is equal to:

$$\omega_{i}=\frac{1}{\mathrm{Var}\left({\bar{Y}}_{i}\right)}=\frac{1}{\left(\frac{\sigma_{wi}^{2}}{n_{i}}+\sigma_{b}^{2}\right)}.$$ (2)

The weight equation, $\omega_{i}=1/\mathrm{Var}\left({\bar{Y}}_{i}\right),$ yields:

$$\omega_{i}\mathrm{Var}\left({\bar{Y}}_{i}\right)=1$$

or

$$\mathrm{Var}\left(\sqrt{\omega_{i}}\;{\bar{Y}}_{i}\right)=1.$$

Generally, all *σ*-values, and consequently the *ω_i_* values are unknown. The $\sigma_{w_{i}}$ can be estimated (as $s_{w_{i}}$) from the replicate measurements. We derive an estimate for $\sigma_{b}^{2}$ and consequently for the *ω_i_* by using the quantity

$$\mathrm{Var}\left(\sqrt{\omega_{i}}{\bar{Y}}_{i}\right)=\frac{\sum_{i=1}^{m}\;\omega_{i}{\left({\bar{Y}}_{i}-\tilde{Y}\right)}^{2}}{m-1}$$

which we equate to unity. Thus we have

$$\frac{\sum_{i=1}^{m}\omega_{i}{\left({\bar{Y}}_{i}-\tilde{Y}\right)}^{2}}{m-1}=1.$$ (3)

Equation (3) is used in “reverse fashion” to estimate the *ω_i_* and $\tilde{Y}$ from the sample data. This is possible if in eq (2), the $\sigma_{w_{i}}$ are estimated from the within-group variability, so that the only unknown is *σ*_b_. Note in eq (3) that *σ*_b_ is embedded within each weight and therefore within $\tilde{Y}$. The estimated $\sigma_{w_{i}}$ and *σ*_b_ can also be used to estimate the standard deviation of the weighted average, which is equal to $1/\sqrt{\sum \omega_{i}}$. Henceforth, we use the symbol *ω_i_* for the sample estimate of *ω_i_*.

The same general reasoning holds for the weighted regression case. The variance of a simple weighted average is replaced by the residual mean square from a weighted least squares regression. For a regression with *m* groups and *p* coefficients the analogue of eq. (3) is

$$\frac{\sum_{i=1}^{m}\omega_{i}{\left({\bar{Y}}_{i}-{\hat{Y}}_{i}\right)}^{2}}{m-p}=1,$$ (4)

where *ω_i_* is given by eq (2) and ${\hat{Y}}_{i}$ is the fitted value.

We now describe the case of a weighted regression with *p* =2. The fitted value ${\hat{Y}}_{i}$, for the *i*th group can be written as follows:

$${\hat{Y}}_{i}=\hat{\alpha }+\hat{\beta }X_{i}$$ (5)

or

$${\hat{Y}}_{i}=\tilde{Y}+\hat{\beta }\left(X_{i}-\tilde{X}\right),$$ (5′)

where $\tilde{X}$ is a weighted average analogous to the weighted average described by eq (1), and $\hat{\alpha }$ and $\hat{\beta }$ are weighted least squares estimates of the coefficients, *α* and *β*. Again, the only unknown is *σ*_b_, which can now be estimated from sample data by use of eq (4).

A *direct* solution for *σ*_b_ in either eq (3) or (4) would be extremely complicated since *ω_i_*, $\tilde{Y}$, and ${\hat{Y}}_{i}$ all contain *σ*_b_. The number of terms *m*, in both equations will vary depending on the number of groups in a particular sample data set. Furthermore, for the regression case, the $\hat{\beta }$ and $\tilde{X}$ also depend on *σ*_b_. Therefore an iterative solution was proposed in reference [1]. This iterative procedure is central to the practical solution of either eq (3) or (4). In order that this paper be self-contained, we briefly review the iterative procedure for the regression case using eq (4) with *p* =2.

## 3. Iteration Procedure

We define the function:

$$F\left(s_{b}^{2}\right)=\sum_{i=1}^{m}\omega_{i}{\left({\bar{Y}}_{i}-{\hat{Y}}_{i}\right)}^{2}-\left(m-2\right).$$ (6)

In view of eqs (2) and (4), the estimate $s_{b}^{2}$ of $\sigma_{b}^{2}$ must be such that $F\left(s_{b}^{2}\right)=0$. For ease of notation let $s_{b}^{2}=\upsilon$. Start with an initial value, *υ*_0_≈0, and calculate an initial set of weights and then evaluate eq (6). In general, $F\left(s_{b}^{2}\right)$ will be different from zero. It is desired to find an adjustment, d*υ*, such that *F*(*υ*_0_*+*d*υ*)=0. Using a truncated Taylor series expansion, one obtains:

$$\begin{array}{r} F\left(\upsilon_{0}+d\upsilon \right)\approx F_{0}+{\left(\frac{\partial F}{\partial \upsilon }\right)}_{0}d\upsilon =0\\ \text{and}\;dv=-F_{0}/{\left(\frac{\partial F}{\partial \upsilon }\right)}_{0}. \end{array}$$

Evaluating the partial derivative in this equation, one obtains:

$$dv=F_{0}/{\left[\sum_{i=1}^{m}\omega_{i}^{2}{\left({\bar{Y}}_{i}-{\hat{Y}}_{i}\right)}^{2}\right]}_{0}.$$ (7)

The adjusted (new) value for υ is:

$$\text{New}\;\upsilon_{0}=\text{Old}\;\upsilon_{0}+d\upsilon .$$ (8)

This new value is now used and the procedure is iterated until dυ is satisfactorily close to zero.

The iterative procedure is easily adapted to the computer. The programming steps are as follows:

1. Evaluate the $s_{w_{i}}$ from the individual groups of data.
2. Start the iteration process with a value of υ_0_ just slightly over zero.
3. Evaluate eq (2) to get estimates of $\omega_{i}$.
4. Fit eq (5) by a weighted least squares regression of ${\bar{Y}}_{i}$ on ${\bar{X}}_{i}$, and get estimates of the ${\hat{Y}}_{i}$.
5. Use eq (6) to evaluate *F*_0_. If *F*_0_<0, then stop the iteration and set υ=0. If not, continue with 6.
6. Use eq (7) to evaluate dυ.
7. If dυ is positive and small enough to justify stopping, then stop. If it is positive, but is not small enough, repeat steps 3–7 [using the new υ_0_ from eq (8)].

The consensus values are the final coefficients of the regression equation. One is also interested in the final $\upsilon =s_{b}^{2}$ value since this is needed to characterize the imprecision of the fit.

For the case of a weighted average [see eq (1)] the above iteration steps are the same, except that in place of step 4, $\tilde{Y}$ is calculated by eq (1), and steps 5 and 6 use $\tilde{Y}$ in place of ${\hat{Y}}_{i}$, and unity is used for the *p* value. The authors have frequently used this procedure for the evaluation of Standard Reference Materials [6].

## 4. Theoretical Extensions

Once one recognizes the between- as well as the within-group component of variance in the evaluation of consensus values, one can begin to consider functional forms for these components. The within-group component can be of *any* form, and can be easily handled since the appropriate sample values of the component are simply substituted into the weights described by eq (2). Thereafter, this component does not affect the iteration procedure. See for example reference [7], where the within component of variance refers to a Poisson process. The between-group component, however, affects the iteration procedure and must be handled more carefully. As an example, consider the case where the between-group component of standard deviation is believed to be a linear function of the level of *X_i_*:

$$\sigma_{b}\approx \gamma +\delta X_{i}.$$ (9)

Let us assume that we have preliminary estimates, *c* and *d* for the *γ* and *δ* coefficients. Suppose further that we wish to adjust the estimated value of the variance by a fixed scale factor, say *υ*′. The desired between-group component of variance is thus:

$$s_{b}^{2}=\upsilon^{\prime }{\left(c+dX_{i}\right)}^{2}.$$ (10)

The weights estimated by eq (2) would then be:

$$\omega_{i}=\frac{1}{\left(\frac{s_{w_{i}}^{2}}{n_{i}}+\upsilon^{\prime }{\left(c+dX_{i}\right)}^{2}\right)}.$$ (2′)

This newly defined weight can be used in the iteration process. The iteration process proceeds as before, but now the adjustable iteration parameter *υ′* is the *multiplier* needed to make eq (4) true, that is, to make it consistent with the sample data sets. The denominator of eq (7) which is used in iteration step 6 for calculating dυ, needs to be slightly modified since the derivative of *F* with respect to υ now contains the function described by eq (10).

$$d\upsilon^{\prime }=\frac{F_{0}}{\sum_{i=1}^{m}\omega_{i}^{2}{\left(c+dX_{i}\right)}^{2}{\left({\bar{Y}}_{i}-{\hat{Y}}_{i}\right)}^{2}}.$$ (7′)

All other steps in the iteration process are the same. The final between-group components of variance will be described by eq (10).

## 5. Example

The iteration process will be used to fit the data of table 1 to a straight line. These are real data taken from a large interlaboratory study for the determination of oxygen in silicon wafers.

A preliminary examination of the data indicates that the within error has a constant standard deviation and that the between error has a standard deviation *proportional* to *X*. Thus, the error structure for the example is given by the equation:

$$\omega_{i}=\frac{1}{\left(\frac{s_{w}^{2}}{n_{i}}+\upsilon X_{i}^{2}\right)},$$

where υ now stands for the product υ*′*d^2^ of eq (2′).

From the replicates, the pooled within standard deviation is readily calculated to be 0.265. The iteration process then yields the following results

$$\begin{array}{l} \;{\hat{Y}}_{i}=-0.0833+3.6085X_{i}\\ \{\begin{array}{l} s_{\text{within}}=0.265\\ s_{\text{between}}=0.0827X_{i}. \end{array} \end{array}$$

**Figure 1:**
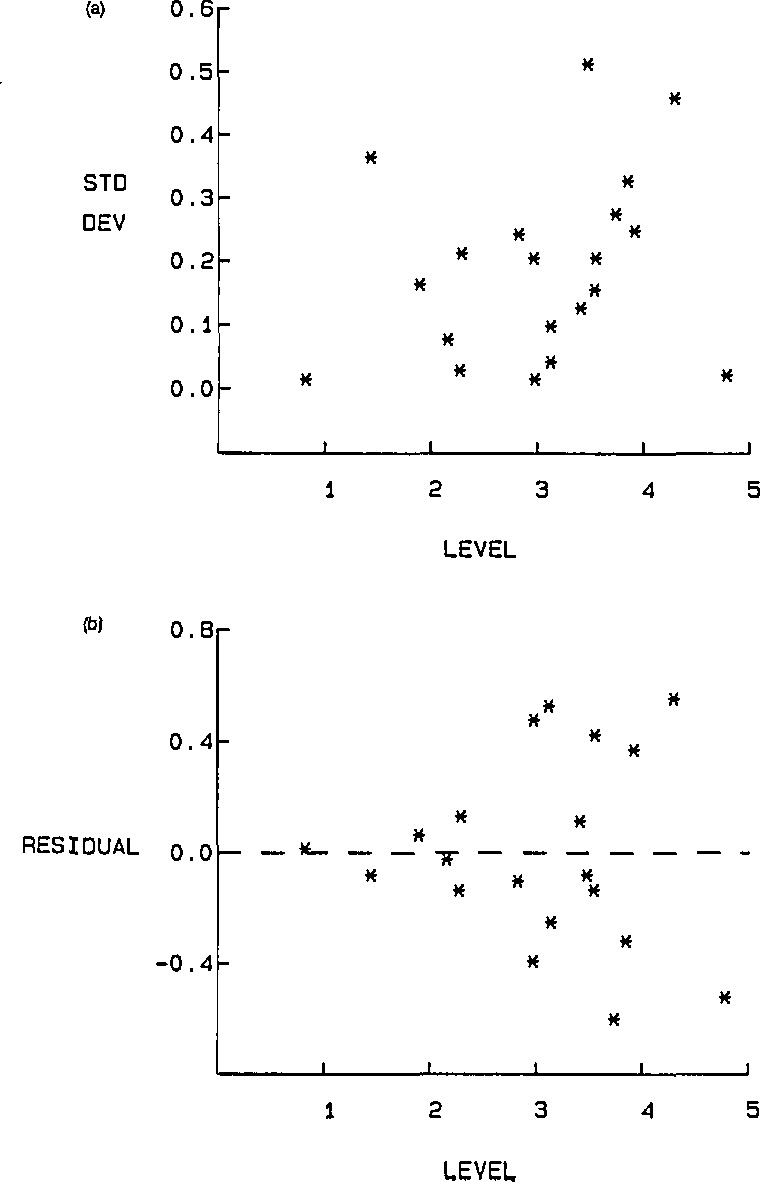
(a) Standard deviations within as a function of *X* (b) residuals of a function of *X*.

Figures 1a and 1b show, respectively, the standard deviations within, and the residuals $\left({\bar{Y}}_{i}-{\hat{Y}}_{i}\right)$, as functions of *X_i_*.

The figures support the assumpions made concerning the nature of the within and between errors.

## Figures and Tables

**Figure 2:**
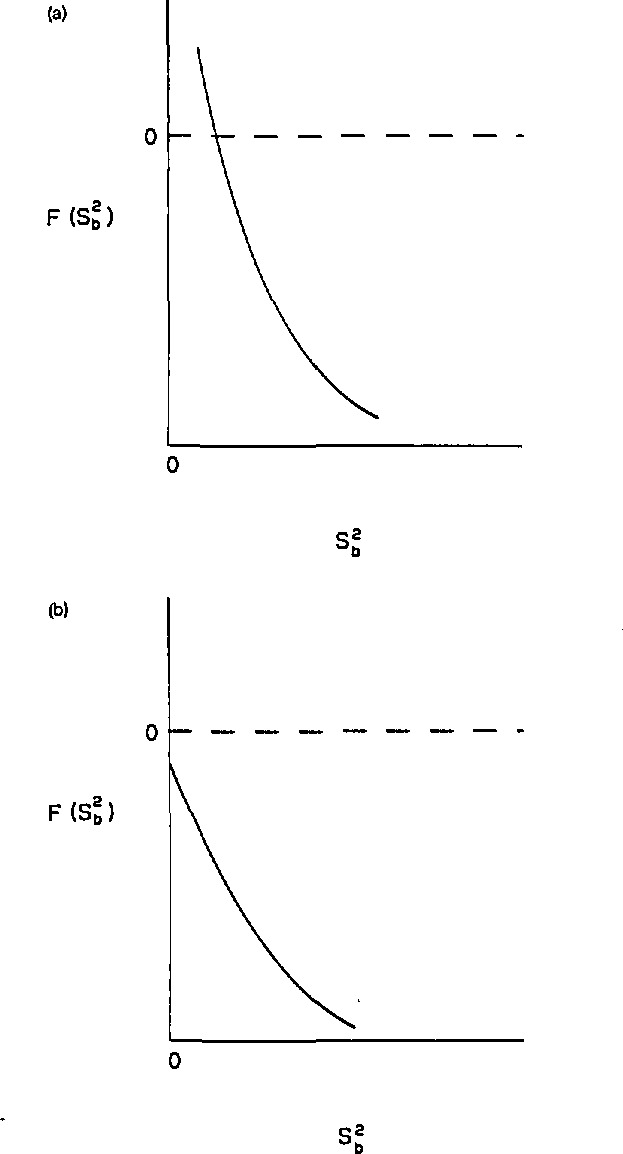
(a) *F* as a function of $s_{b}^{2}$ where *F* has a positive root for $s_{b}^{2}$ (b) *F* as a function of $s_{b}^{2}$ where *F* does not have a positive root for $s_{b}^{2}$.

**Table 1:** Data used in example of iteration process

*X*	*Y*_1_	*Y*_2_	*Y*_3_
0.806	2.83	2.85	
1.429	4.62	5.35	5.01
1.882	6.89	6.66	
2.140	7.56	7.67	
2.256	7.94	7.90	
2.279	8.42	8.12	
2.814	10.04	9.70	10.17
2.957	10.34	10.05	
2.961	11.09	11.07	
3.108	11.63	11.69	
3.124	10.87	11.01	
3.403	12.40	12.22	
3.466	11.94	12.17	12.92
3.530	12.63	12.41	
3.543	12.98	13.27	
3.724	12.95	12.56	
3.836	13.07	13.69	13.56
3.902	14.54	14.19	
4.280	15.59	16.24	
4.770	16.62	16.59	

## 6. Appendix

### 6.1 Sketch of Proof of Convergence

The general functional form of the *F* of eq (6) is as shown in figure 2a or 2b. It is because of the nature of these forms that convergence always occurs.

If the functional form of *F* is as shown in figure 2a, the previously described iterative procedure is used to determine the $s_{b}^{2}$ satisfying the equation $F\left(s_{b}^{2}\right)=0$. If an initial estimate of $s_{b}^{2}$ is chosen that is very slightly above zero, then convergence of the iteration process always occurs. This is a result of the fact that the first derivative of the function *F* with respect to $s_{b}^{2}$ is negative, and the second derivative is positive. This means that each iteration will undershoot, since the iteration process extrapolates the slope of the *F* curve at the current $s_{b}^{2}$ estimate to the *F*=0 value. Since each new iteration estimate of $s_{b}^{2}$ is the abscissa value of the intersection of the tangent line with the *F*=0 horizontal line, the iteration process will never overshoot and convergence is obtained.

If the form is that of figure 2b, then there will be no positive solutions for the $s_{b}^{2}$ that is associated with the function *F*. This represents a situation where the variability between the sample groups is less than that expected from the variability within the sample groups. For this situation *F*_0_ is negative and $s_{b}^{2}$ is set to zero (see iteration step 5).

The proof regarding the signs of the first and second derivatives of *F* with respect to $s_{b}^{2}$ follows. The simple *regression case* will be considered, with the $s_{b}^{2}=\upsilon$ being constant. (The extension to the variable $s_{b}^{2}$ case is straightforward.)

### 6.2 Proof That the First Derivative of *F* is Negative

An examination of eqs (1), (2), (5′). and (6) shows *ω_i_* and ${\hat{Y}}_{i}$ to be functions of $s_{b}^{2}$. Equation 5′ also indicates that $\tilde{Y}$, $\tilde{X}$, and *β* are functions of $s_{b}^{2}$. We start with the first derivative of the *F* of eq (6)

$$\frac{dF}{d\upsilon }=\frac{d\left(\sum_{i=1}^{m}\omega_{i}{\left({\bar{Y}}_{i}-{\hat{Y}}_{i}\right)}^{2}-\left(m-2\right)\right)}{d\upsilon }$$

The derivative of *ω_i_* will frequently be encountered in the following material. At this point, it will be convenient to note its value:

$$\frac{d\omega_{i}}{d\upsilon }=-\omega_{i}^{2}.$$

Continuing, and making use of eq (5′):

$$\begin{array}{l} \frac{dF}{d\upsilon }=-\sum_{i=1}^{m}\omega_{i}^{2}{\left({\bar{Y}}_{i}-{\hat{Y}}_{i}\right)}^{2}\\ \;-2\left(\frac{d}{d\upsilon }\left({\bar{Y}}_{i}-\hat{\beta }\tilde{X}\right)\right)\sum_{i}\omega_{i}\left({\bar{Y}}_{i}-{\hat{Y}}_{i}\right)\\ \;-2\left(\frac{d\hat{\beta }}{d\upsilon }\right)\sum_{i}\omega_{i}X_{i}\left({\bar{Y}}_{i}-{\hat{Y}}_{i}\right). \end{array}$$ (A1)

The last two terms of eq (A1) each contain summations that are equal to zero, so these terms drop out. Next, an examination of the remaining term shows that each product is a positive square, and that the summation is preceded by a minus sign. Thus, the first derivative is negative.

### 6.3 Proof That the Second Derivative of *F* is Positive

The evaluation of the second derivative is involved and only an outline of the steps is presented.

$$\begin{array}{r} \frac{d^{2}F}{d\upsilon^{2}}=\frac{d}{d\upsilon }\left[-\sum_{i=1}^{m}\omega_{i}^{2}{\left({\bar{Y}}_{i}-{\hat{Y}}_{i}\right)}^{2}\right],\\ \frac{d^{2}F}{d\upsilon^{2}}=-2\sum_{i}\omega_{i}^{2}\left({\bar{Y}}_{i}-{\hat{Y}}_{i}\right)\frac{d\left({\bar{Y}}_{i}-{\hat{Y}}_{i}\right)}{d\upsilon }\\ +2\sum_{i}\omega_{i}^{3}{\left({\bar{Y}}_{i}-{\hat{Y}}_{i}\right)}^{2}, \end{array}$$ (A2′)

where

$$\begin{array}{r} \frac{d\left({\bar{Y}}_{i}-{\hat{Y}}_{i}\right)}{d\upsilon }=-\frac{d\tilde{Y}}{d\upsilon }+\hat{\beta }\frac{d\tilde{X}}{d\upsilon }\\ -\left(X_{i}-\tilde{X}\right)\frac{d\hat{\beta }}{d\upsilon }. \end{array}$$ (A3)

Evaluation of the first two derivatives on the r.h.s. of eq (A3) yields:

$$\begin{array}{r} \frac{d\tilde{X}}{d\upsilon }=-\sum_{i}\frac{\omega_{i}^{2}}{W}\left(X_{i}-\tilde{X}\right)\;\text{and}\\ \frac{d\tilde{Y}}{d\upsilon }=-\sum_{i}\frac{\omega_{i}^{2}}{W}\left({\bar{Y}}_{i}-\tilde{Y}\right), \end{array}$$ (A4)

where *W*≡Σ *ω_i_*.

Evaluation of the last derivative of eq (A3) yields:

$$\begin{array}{c} \frac{d\hat{\beta }}{d\upsilon }=\frac{d\left[\frac{\sum_{i}\omega_{i}\left(X_{i}-\tilde{X}\right){\bar{Y}}_{i}}{\sum_{i}\omega_{i}{\left(X_{i}-\tilde{X}\right)}^{2}}\right]}{d\upsilon }=\\ \sum_{t=1}^{m}\left[\frac{d\left[\frac{\sum_{i}\omega_{i}\left(X_{i}-\tilde{X}\right){\bar{Y}}_{i}}{\sum_{i}\omega_{i}{\left(X_{i}-\tilde{X}\right)}^{2}}\right]}{d\omega_{t}}\cdot \frac{d\omega_{t}}{d\upsilon }\right], \end{array}$$ (A5′)

where

$$\begin{array}{c} \frac{d\sum_{i}\omega_{i}\left(X_{i}-\tilde{X}\right){\bar{Y}}_{i}}{d\omega_{t}}=\left(X_{t}-\tilde{X}\right)\left({\bar{Y}}_{t}-\tilde{Y}\right),\\ \frac{d\sum_{i}\omega_{i}{\left(X_{i}-\tilde{X}\right)}^{2}}{d\omega_{t}}={\left(X_{t}-\tilde{X}\right)}^{2}, \end{array}$$

and

$$\frac{d\left[\frac{\sum_{i}\omega_{i}\left(X_{i}-\tilde{X}\right){\bar{Y}}_{i}}{\sum_{i}\omega_{i}{\left(X_{i}-\tilde{X}\right)}^{2}}\right]}{d\omega_{t}}=\frac{\left(X_{t}-\tilde{X}\right)\left({\bar{Y}}_{t}-{\hat{Y}}_{t}\right)}{\sum_{i}\omega_{i}{\left(X_{i}-\tilde{X}\right)}^{2}}.$$

Reassembling eq (A5′):

$$\frac{d\hat{\beta }}{d\upsilon }=\frac{\sum_{t}\omega_{t}^{2}\left(X_{t}-\tilde{X}\right)\left({\bar{Y}}_{t}-{\hat{Y}}_{t}\right)}{\sum_{i}\omega_{i}{\left(X_{i}-\tilde{X}\right)}^{2}}$$ (A5)

At this point the running index *t* can be conveniently changed back to the index *i*. Finally, the substitution of eq (A5) into (A3), and then eq (A3) into (A2′) yields:

$$\begin{array}{c} \frac{d^{2}F}{d\upsilon^{2}}=2[\sum_{i}\omega_{i}^{3}{\left({\bar{Y}}_{i}-{\hat{Y}}_{i}\right)}^{2}-\frac{1}{W}{\left[\sum_{i}\omega_{i}^{2}\left({\bar{Y}}_{i}-{\hat{Y}}_{i}\right)\right]}^{2}\\ -\frac{{\left[\sum_{i}\omega_{i}^{2}\left(X_{i}-\tilde{X}\right)\left({\bar{Y}}_{i}-{\tilde{Y}}_{i}\right)\right]}^{2}}{\sum_{i}\omega_{i}{\left(X_{i}-\tilde{X}\right)}^{2}}]. \end{array}$$ (A2′ ′)

This second derivative eq (A2′ ′) is a residual weighted sum of squares from regression. To see this, let

$$Z_{i}\equiv \omega_{i}\left({\bar{Y}}_{i}-{\hat{Y}}_{i}\right)$$ (A6)

and substitute into eq (A2′ ′):

$$\begin{array}{c} \frac{d^{2}F}{d\upsilon^{2}}=2[\sum_{i}\omega_{i}Z_{i}^{2}-\frac{1}{W}{\left[\sum_{i}\omega_{i}Z_{i}\right]}^{2}\\ \begin{array}{c} -\frac{{\left[\sum_{i}\omega_{i}\left(X_{i}-\tilde{X}\right)Z_{i}\right]}^{2}}{\sum_{i}\omega_{i}{\left(X_{i}-\tilde{X}\right)}^{2}}]\\ =2\left[\sum_{i}\omega_{i}{\left(Z_{i}-\tilde{Z}\right)}^{2}-\frac{{\left[\sum_{i}\omega_{i}\left(X_{i}-\tilde{X}\right)Z_{i}\right]}^{2}}{\sum_{i}\omega_{i}{\left(X_{i}-\tilde{X}\right)}^{2}}\right] \end{array} \end{array}$$

The first term on the r.h.s. is the “total” weighted sum of squares of *Z*. The second term is the weighted sum of squares for the regression of *Z* on *X*. Therefore the difference between the two terms is a “residual” sum of squares:

$$\frac{d^{2}F}{d\upsilon^{2}}=2\sum_{i}\omega_{i}{\left(Z_{i}-{\hat{Z}}_{i}\right)}^{2},$$ (A2)

where ${\hat{Z}}_{i}$ is the fitted value of *Z_i_* in a weighted regression of *Z* on *X*. Thus, the second derivative is positive for *ω_i_*>0. The iteration process therefore will never overshoot, and convergence is always assured.

### 6.4 Extensions

The extension of the convergence proof to the variable $s_{b}^{2}$ case is very similar to that given above. Two basic changes are needed. These changes, which introduce a function of *X_i_*, are in the derivative of *ω_i_*, and in the definition of *Z_i_*.

$$\frac{d\omega_{i}}{d\upsilon }=-g\left(X_{i}\right)\omega_{i}^{2}$$

and

$$Z_{i}=\omega_{i}g\left(X_{i}\right)\left({\bar{Y}}_{i}-{\hat{Y}}_{i}\right).$$

Equation (10) represents an example of a variable $s_{b}^{2}$. For that case,

$$g\left(X_{i}\right)={\left(c+dX_{i}\right)}^{2}.$$

The reader may note that the new *Z_i_* contains *X_i_*, and that *Z_i_* is regressed on *X_i_*. The argument does not require that this regression “make sense”, only that the sum of squares can be partitioned by a regression process. Again, convergence is obtained.

The weighted average is a special and simple application of the weighted regression case.
